# Mesenchymal stromal cells induced regulatory B cells are enriched in extracellular matrix genes and IL-10 independent modulators

**DOI:** 10.3389/fimmu.2022.957797

**Published:** 2022-09-14

**Authors:** Sergio G. Garcia, Noelia Sandoval-Hellín, Marta Clos-Sansalvador, Laura Carreras-Planella, Miriam Morón-Font, Dolores Guerrero, Francesc E. Borràs, Marcella Franquesa

**Affiliations:** ^1^ REMAR-IGTP Group, Germans Trias i Pujol Research Institute (IGTP) & Nephrology Department, University Hospital Germans Trias i Pujol (HUGTiP), Can Ruti Campus, Badalona (Barcelona), Catalonia, Spain; ^2^ Department of Cell Biology, Physiology and Immunology, Autonomous University of Barcelona, Bellaterra, Spain; ^3^ Otorhinolaryngology Department, Hospital Universitari Germans Trias i Pujol, Badalona, Spain

**Keywords:** B cell, immunomodulation, mesenchymal stromal cell, regulatory B cell, IL-10

## Abstract

Regulatory B cells (Breg) are essential players in tolerance and immune homeostasis. However, lack of specific Breg markers limit their potential in clinical settings. Mesenchymal stromal cells (MSC) modulate B cell responses and are described to induce Breg *in vitro.* The aim of this work was to characterize MSC induced Breg (iBreg) and identify specific Breg biomarkers by RNAseq. After 7-day coculture with adipose tissue-derived MSC, B cells were enriched in transitional B cell populations, with increased expression and secretion of IL-10 and no TNFα. In addition, iBreg showed potential to modulate T cell proliferation at 2 to 1 cell ratios and their phenotype remained stable for 72h. RNAseq analysis of sorted IL-10 positive and negative iBreg populations identified over 1500 differentially expressed genes (DEG) among both populations. Analysis of biological processes of DEG highlighted an enrichment of immune regulation and extracellular matrix genes in IL-10^-^ iBreg populations, while IL-10^+^ iBreg DEG were mostly associated with cell activation. This was supported by T cells modulation assays performed in the presence of anti-IL-10 neutralizing antibodies showing the non-essential role of IL-10 in the immunomodulatory capacity of iBregs on T cells. However, based on RNAseq results we explored the role of TGF-β and found out that it plays a major role on iBreg induction and iBreg immunomodulatory properties. Therefore, we report that MSC induce B cell populations characterized by the generation of extracellular matrix and immune modulation independently of IL-10.

## Introduction

B lymphocytes are the highlight of the adaptive humoral response mainly by their production of antigen-specific immunoglobulins (Ig) or antibodies (1, 2). Yet, B lymphocytes have essential roles besides antibody production, including antigen presentation and co-stimulation, and the production of secretory mediators (i.e. cytokines), that modulate cellular responses (3, 4).

A specific subset of B lymphocytes, known as regulatory B cells (Breg), can modulate the immune response by suppressing T cell activation and proliferation, inducing differentiation of regulatory T cells, and producing immunomodulatory factors such as IL-10 (5). Breg are essential in the maintenance of immune homeostasis and immunological tolerance, but despite extensive discussion and research done in recent years (5–8), several drawbacks including the lack of unique defining markers, and the heterogeneity across experimental settings and animal models are undermining their therapeutic potential. Regarding the first, the general consensus on human studies is that IL-10 identifies Bregs *in vivo* and is crucial to exert their immunomodulatory role. On the second, the term Breg may include several B cell subsets such as transitional B cell (8–10), GMZB^+^ B cells (11–13), or CD1d^+^ B cells (14–16), most of them with immunoregulatory effect. In deep contrast to their T regulatory counterparts (17), no transcription factors nor surface markers have been unequivocally associated to human Breg.

Characterization of Breg is a priority for their future clinical application. Breg are limitedly represented in peripheral circulation. Thus, Breg characterization is mostly performed by culturing B cells *in vitro* using a plethora of B cell activating molecules, such as anti-BCR antibodies, CD40L, IL-21, CpG, or LPS, among others. In recent years, mesenchymal stromal/stem cells (MSC) have been also described to induce Breg *in vitro* (18–21).

MSC are immunomodulatory non-hematopoietic cells that can be isolated from several tissues, that hold stem cell properties and can be phenotypically defined by a set of minimal criteria (22, 23). MSC are widely studied for their broad clinical potential in immune modulation, wound healing, and tissue repair. In recent years, MSC have been described to interact with several immune cell types, including B cells, reducing their terminal differentiation, inhibiting antibody production, and promoting their differentiation to Breg (18–21). Taking into account their role in B cell modulation, MSC constitute an optimal candidate to induce and expand Breg *in vitro* for downstream application.

In the present study, we use a multicomponent coculture system to expand and study *in vitro* MSC-induced Breg (iBreg). First, we characterized iBreg according to surface markers, IL-10/TNFα expression, and immunomodulatory capabilities. Secondly, to search for new Breg cell markers and increase knowledge on Breg physiology, we purified IL-10^+^ and IL-10^-^ iBreg to perform transcriptomic characterization by RNA-seq and identify differentially expressed genes. Finally, we assessed the role of IL-10 as a key molecule of Breg’s immunomodulatory capacity. The results of this study show that MSC induce regulatory B cells *in vitro* that were able to suppress T cell proliferation, independently of IL-10 action. Characterization by transcriptomic analysis of Breg IL-10 positive and negative populations showed an enrichment of immunomodulation- and extracellular matrix organization-related genes in IL-10^-^ cells. This set of genes could become potential Breg biomarkers, and highlight the importance of IL-10^-^ Breg populations for future Breg and MSC studies.

## Materials and methods

### Mesenchymal stem cell collection and culture

MSC from subcutaneous adipose tissue were obtained from healthy donors at “Hospital Germans Trias i Pujol”, MSC multilineage differentiation potential and surface markers were tested (**Supplementary Figure 1**) upon culture to meet the minimal criteria for MSC identification (22) as described in [ (19, 24)]. Informed consent was obtained from all subjects, and the study protocol conformed to the principles outlined in the Declaration of Helsinki. Cells were frozen at -190°C until further use. To perform the experiments cells were cultured in 175 cm^2^ flasks at 37°C, 5% CO2, and 95% humidity, in 20 mL of minimum essential medium-α (Lonza, Verviers, Belgium) supplemented with 1% penicillin (100 IU/ml, Cepa S.L., Madrid, Spain) and streptomycin (100 mg/ml, Normon Laboratories S.A., Madrid, Spain), 1% L-glutamine (Sigma Aldrich, St. Louis, MO, USA), and 10% heat-inactivated fetal bovine serum (Lonza). Medium was renewed every 2-4 days and flasks were examined by optical microscopy and when 90% confluence was reached, cells were detached with trypsin-EDTA (Lonza) at a concentration of 0.05% during 5 minutes at 37°C. Passages 2 to 8 were used for experiments. To asses surface marker expression, MSC were labelled for flow cytometry analysis with the following antibodies: CD90-PE-Cy7 (BioLegend, clone 5E10, San Diego, CA, USA), CD105-FITC (ImmunoTools, clone MEM-226SJ25C1, Friesoythe, Germany), CD73-PE (BD Pharmingen, clone AD2), CD45-PerCP (ImmunoTools, clone HI30), HLD-DR-APC-H7 (BD Biosciences, clone L243).

### B cell isolation from human tonsils

To isolate B cells, tonsils were obtained from patients that underwent tonsillectomy at the Hospital Universitari Germans Trias i Pujol (HUGTiP) to be anonymously used for research purposes. All participants and/or their legal tutor gave written informed consent to participate. The study protocol followed the principles of the Declaration of Helsinki and was approved by the Clinical Research Ethics Committee of our institution (Comitè Ètic d’Investigació Clínica, Refs CEIC: PI-16-056 from 5 May 2016). Tonsils were mechanically disrupted and filtered through a 70 μm cell strainer, to then isolate mononuclear cells by density gradient separation with Ficoll-Paque (GE Healthcare, Uppsala, Sweden). Mononuclear cells were frozen in 90% heat-inactivated fetal bovine serum (Lonza) and 10% Dimethyl sulfoxide (DMSO) and stored at -190°C until further use. Upon thawing, resting B cells were isolated by negative selection by labelling anti-CD43 magnetic beads (Miltenyi Biotec GmbH, Bergisch Gladbach, Germany). Purity was confirmed by Flow cytometry (FACS Canto II, BD Bioscience, San Jose CA), labelling cells with the following antibodies; 7AAD (BD Biosciences), CD3-PE (BD Biosciences, Clone UCHT1) and CD19-BV510 (BD Horizon, clone SJ25C1), ensuring CD19 purities above 95%.

### MSC and B cell coculture

For the generation of iBreg, B cells and MSC were co-cultured in 96-well flat bottom plates for 7 days. 50000 B cells and 5000 MSC were seeded per well. B cells were cultured in 200 μL of IMDM culture medium (Lonza) supplemented with 1% penicillin (100 IU/ml, Cepa S.L., Madrid, Spain) + streptomycin (100 mg/ml, Normon Laboratories S.A. Madrid, Spain), 1% L-glutamine (Sigma- Aldrich), and 10% heat-inactivated fetal bovine serum (Lonza). Cells were stimulated with a combination of monoclonal antibodies that mimic T helper cell action (T-cell like: TCL); 10 μg/mL of F(ab)_2_ anti-IgM (Jackson, ImmunoResearch laboratories) 10^3^ IU of IL-2 (Peprotech, London, UK) and 2.5 ug/mL of CD40 agonist (Bioxcell, Clone G28.5). For the generation of activated B cells, 50000 cells were cultured in 96-well round bottom plates with no MSC. A minimum of 2 MSC donors and 3 Tonsil donors were used for each experiment.

### iBreg characterization

To asses surface marker expression, iBreg cultures were labelled for flow cytometry analysis with the following antibodies: IgD-APC-Cy7 (BioLegend, clone IA6-2, San Diego, CA, USA), CD19-BV510 (BD Horizon, clone SJ25C1), CD24-APC (Invitrogen, clone eBioSN3), CD27-PE-Cy7 (Invitrogen, clone 0323), CD38-PE (Invitrogen, clone HB7) and 7AAD (BD Pharmingen).

To asses cytokine production, supernatants were collected and kept at -20°C for further quantification of IL-10 and TNFα in two independent ELISA tests, both according to manufacturer’s instructions (U-Cytech, Utrecht, The Netherlands). Standards were provided by the kit and a standard curve was prepared from 200 to 3 pg/mL. Samples were measured at 450 nm using an absorbance plate reader (Varioskan).

To quantify IL-10 and TNFα intracellular expression in B cells, we performed intracellular staining of both cytokines in independent tubes, labelling each B cell tube with IL-10-PE (BD Pharmingen, Clone JES3-19F1) or TNFα-PE (BD Pharmingen, Clone MAb11) using IntraStain kit (Dako Denmark). Monensin (5 mM, BD Biosciences) 1/6000 solution was added twelve hours before performing the intracellular staining procedure to block cell protein secretion. In some cases, for PMA and ionomycin samples, 4h before intracellular staining samples were stimulated with 50 ng/mL of PMA (Sigma) and 500 ng/mL of Ionomycin (Merk, Sigma-Aldrich) and Monensin 1/6000 dilution was added at the same time to the samples. Blank samples without fluorochrome-conjugated antibodies were prepared as a control for cytometry. Intracellular staining was performed following manufacturer’s instructions.

To study iBreg phenotypical stability, iBreg were collected at day 7 from MSC-B cell cocultures and cultured for an additional 72h period in 96w round bottom culture plates in the presence of different B cell stimulation molecules: TCL stimuli as defined in the “MSC and B cell coculture” section, 10 µg/mL LPS (*E. coli*, O111:B4, Sigma-Aldrich) or 10 µg/mL CpG (oligodeoxynucleotide-2006, Invivogen). B cells were analyzed by flow cytometry using the previously defined panel at 24h, 48h and 72h.

### T cell proliferation assays

To test iBreg immunomodulatory capacity, purified CD43- B cells were co-cultured with MSCs. After 7 days of co-culture their regulatory potential was tested on T cell proliferation. T cells were isolated from tonsillar PBMCs to perform autologous assays by magnetic separation using HLA kit according to manufacturer’s protocol for lymph nodes samples. After isolation, purity was confirmed by flow cytometry (FACS Canto II, BD Bioscience, San Jose CA), labelling cells with the following antibodies; 7AAD (BD Pharmingen), CD3-PE (BD Pharmingen, Clone UCHT1) and CD19-BV510 (BD Horizon, clone SJ25C1). Purities were above 85% in all samples.

T cells were labelled with CFSE (5 mM) 1/6000 solution in PBS for 10 min at RT and stimulated with αCD2/CD3/CD28 beads (Miltenyi Biotec, T Cell Activation/Expansion Kit, human). 50000 T cells were cultured in 96-well round bottom plates in 200 μL of IMDM culture medium (Lonza) supplemented with 1% penicillin (100 IU/ml, Cepa S.L., Madrid, Spain) + streptomycin (100 mg/ml, Normon Laboratories S.A.,Madrid, Spain), 1% L-glutamine (Sigma- Aldrich), and 10% heat-inactivated fetal bovine serum (Lonza) with the following ratios of T to iBreg cells: 1:2, 1:1, 2:1, 5:1, 10:1, and 50:1. Proper controls were added for the experiments, no CFSE labelling, no proliferation stimuli, and no iBreg. 4 days after, proliferation was assessed by Flow cytometry. Samples were labelled with CD19-BV510 (BD Horizon, clone SJ25C1) to exclude B cells from the analysis. For experiments assessing IL-10 role in T cell modulation, we used anti-IL-10 neutralizing antibodies (BD Biosciences) at concentrations of 10 and 20 μg/mL. To inhibit TGFBR1, the inhibitor Ly-364947 (MedChemExpress, Sweden) was added to wells at 2 μM and 5 μM as described in the literature (25, 26). IL-10 neutralization capacity was confirmed by ELISA (U-Cytech). Recombinant IL-10 (Peprotech) was measured at 50 and 100 pg/mL. For neutralized samples, 10 or 20 μg/mL of anti-IL10 antibody was added to IL-10 samples and incubated for 30 min at RT before adding samples to the ELISA wells (**Supplementary Figure 2**).

### Il-10 Capture assay and cell sorting

To isolate IL-10 positive and negative B cells, we performed flow cytometry cell sorting (BD Aria II) by labelling iBreg samples with a modified version of the IL-10 capture assay (Miltenyi Biotec GmbH, Bergisch Gladbach, Germany). 4 hours prior to collecting B cells, 0.01 μL of PMA (Sigma) and 0.2 μL of Ionomycin (Merk, Sigma-Aldrich) were added to the stimulated samples to boost cytokine production on B cells. In brief, after collecting iBreg, cells were washed with cold commercial MACS buffer (Miltenyi Biotec GmbH, Bergisch Gladbach, Germany) and resuspended in 80 μL of cold IMDM complete medium. Then, B cells were labelled with 20 μL of IL-10 catch reagent per 10 million cells, with an incubation of 5 min on ice. Next, we added 10 mL of warm medium and incubated at 37°C for 45 min under constant agitation to promote IL-10 secretion. Cells were washed with cold MACS buffer and resuspended in 80 μL cold MACS buffer. Next, cells were labelled with 20 μL of IL-10-PE (Biologend, clone JES3-9D7) detection antibody per 10 million cells, incubating for 10 min on ice. At this point, instead of following manufacturer’s instructions to perform magnetic separation, we performed Flow cytometry associated cell sorting. We sorted cells in two groups according to high-expressing IL-10^+^ and IL-10^-^ iBreg.

### RNAseq and data analysis

RNA extraction was performed using Norgen Single Cell RNA purification kit (Norgen Biotech, Cat. 51800) according to manufacturer’s instructions, with no β-mercaptoethanol and performing DNase I treatment (Qiagen RNase-free DNase I) for 15 min with a mix of 10 μL of DNase I with 70 μL of Qiagen’s RDD buffer added directly to the column. All RNA samples were quantified and their quality assed using Agilent 2200 Bioanalyzer System by the IGTP Genomics platform, with a High Sensitivity RNA ScreenTape^®^ analysis. All samples had high RNA integrity values (RIN ≥ 9). Ribosomal RNA was depleted using NEB Next rRNA Depletion Kit (Human/Mouse/Rat) and RNA libraries generated by the NEB Next Ultra II Directional RNA Library Prep Kit. Libraries’ quality was assessed on a Tape Station using a “High Sensitivity D1000 Screen Tape” (Agilent) and quantified by fluorometry (Quantus, Promega) and qPCR (KAPA library quantification kit, Light Cycler 480). Final quantification of each library was obtained from the quantifications derived from fluorometry and qPCR corrected by the fragment size observed in capillary electrophoresis, and used to prepare an equimolar mixture of all libraries. Libraries were denatured with sodic hydroxide reaching a 20 pM concentration library pool. Samples were loaded into an Illumina HighSeq sequencer, obtaining 20-25M reads per sample. Raw RNA-seq data quality was check by FASTQC. Paired-end reads for each sample were quantified *via* alignment to version 38 of the Ensembl annotation of the human genome using HISAT2 (27, 28). The DESeq2 package (29) was used to normalize and analyze differential gene expression between groups, IL-10^+^ and IL-10^-^ cells. Batch effect was corrected during differential gene expression analysis, considering biological variability from MSC and B cell donor. Genes with a Benjamini–Hochberg false-discovery rate (FDR) inferior to 0.01 and a foldchange (FC) superior to 2 were considered significantly differentially expressed. Gene ontology (GO), chord diagrams and volcano plots were generated in R using *clusterProfiler* (30, 31)*, circlize* (32) and *ggplot2* (33), respectively. Only GO categories with an FDR under 10% were represented. Heatmaps were generated in R using Heatmap3 package (34). String (35) software was used to analyze gene interaction networks based on their protein interactions, with a special interest for functional clusters of biological processes.

### qPCR

To analyze specific gene expression, whole RNA content was isolated from cells using the RNAeasy Mini kit (Qiagen). cDNA was obtained from RNA samples using random hexamers and the One-step RT-PCR kit (BioRad laboratories) according to the manufacturer’s protocol using Applied biosystems 2729 Thermal Cycler. Analysis of gene expression was performed using the primer sequences indicated in **Supplementary Table 3**. qPCR reaction was performed and analyzed on the LightCycler 480 II-Real-Time PCR System (Roche) using “Master Mix PowerUp SYBR green” kit (Thermo Fisher Scientific). Each sample was analyzed in triplicate. 18S gene was used as endogenous reference to quantify relative expression of each gene by the difference in threshold cycle method, 2(−ΔΔCt) and represented as the log2FC.

## Results

### MSC induce B cells enriched in transitional B cell populations with increased IL-10 secretion

**Figure 1:**
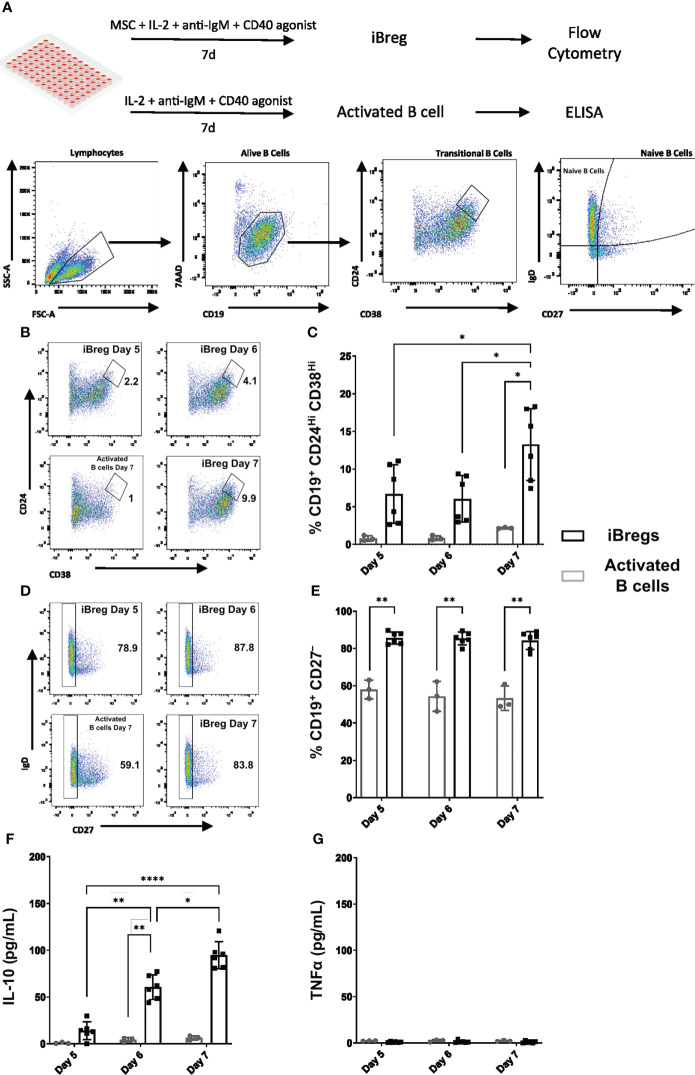
MSC induce regulatory B cells enriched in transitional B cell phenotypes and secrete IL-10. **(A)** Graphical protocol for the generation of *in vitro* induced regulatory B cells (iBreg) and activated B cells and downstream analysis including gating strategy of flow cytometry samples. **(B)** Representative FACS plots showing the percentage of Transitional B (CD19^+^CD24^Hi^CD38^Hi^) cells from alive B cells (CD19^+^7AAD^-^). **(C)** Summarized data for the frequency of Transitional B cells, showing a significant increase at day 7 **(D)** Representative FACS plots showing the percentage of naïve B cells (CD19^+^CD27^-^) from alive B cells (CD19^+^7AAD^-^). **(E)** Summarized data for the frequency of Naïve B cells showing a significant increase in the presence of MSC. **(F)** Supernatant cytokine quantification by ELISA of IL-10. IL-10 production increased after day 5, with a peak production observed at day 7. **(G)** Supernatant cytokine quantification by ELISA of TNFα. TNFα production was absent in iBreg and activated B cells. Data shows at least three independent experiments in each group. Cells from 2 MSC donors and 3 Tonsil donors were used. Error bars represent SD. Two-way ANOVA was performed to determine statistical significance. *p < 0.05, **p < 0.01, ****p < 0.0001.

Human studies have shown that Breg are mostly found among transitional naïve population expressing IL-10 in the absence of proinflammatory cytokines. Concordantly, to study Breg induction in our system, we analyzed *in vitro* induced regulatory B cells (iBreg) after 7-day coculture of tonsillar B cells with MSC in the presence of a T-cell like stimuli (TCL) (**Figure 1A**). We assessed by flow cytometry transitional B cell and naïve/memory phenotypes, and by ELISA, we quantified IL-10 and TNFα secretion.

After 7 days in coculture with MSC, B cells were enriched in transitional B cells (CD19^+^ CD24^Hi^ CD38^Hi^, 14%), and significantly increased naïve B cell populations (CD19^+^ CD27^-^, 84%), compared to activated B cells (2.26% and 53.3% respectively) (**Figures 1B-E**). Kinetic study of the induction of Transitional B cell populations, showed that they were significantly increased over time peaking at day 7, while naïve B cell populations remained stable in the presence of MSC. These Transitional B cells were predominantly of a naïve phenotype, as over 90% were identified as naïve B cells (CD19^+^ IgD^+^ CD27^-^) (**Supplementary Figure 3**). IL-10 secretion increased after day 5, with a peak average production at day 7 of 95 pg/mL, (**Figure 1F**). Moreover, no TNFα was detected in supernatants (**Figure 1G**), indicating no production of pro-inflammatory mediators by iBreg. By contrast, activated B cells did not show any production of IL-10 nor TNFα after day 5, suggesting a lack of long-term cellular stimulation in the absence of MSC.

Based on these observations, and according to surface markers and cytokine expression, we stablished day 7 as the reference timepoint for downstream experiments involving iBreg analysis due to transitional B cell enrichment and maximum IL-10 secretion in all samples.

### iBreg inhibit T cell proliferation and are stable after MSC coculture

**Figure 2:**
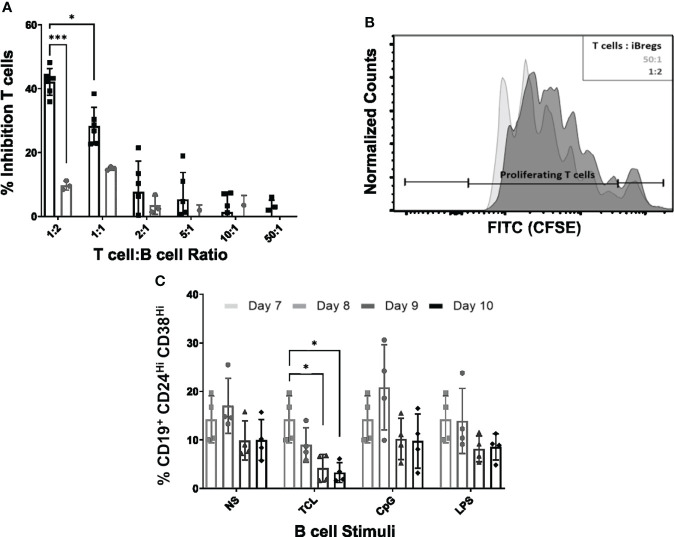
iBreg suppress T cell proliferation. iBreg stability was analyzed after day 7 in the absence of MSC showing high stability. **(A)** T cell proliferation assay showing percentage of MFI (median fluorescence intensity) reduction for each condition compared to stimulated T cells. iBreg inhibited T cell proliferation at high B to T cell ratios **(B)** Histogram representing CFSE expression on T cells **(C)** Graph represents iBreg transitional B cell populations in stability assays. Figure represents transitional values grouped by stimuli condition. iBreg were for the most parts stable under different stimuli after coculture with MSC. Only significantly different condition was TCL stimuli, decreasing the percentage of transitional B cell. Data shows at least three independent experiments in each group. Cells from 2 MSC donors and 3 Tonsil donors were used. Error bars represent SD. Two-way ANOVA was performed to determine statistical significance. *p < 0.05, ***p < 0.001.

In order to validate the immunomodulatory role of enriched Transitional B cell and Breg induction *in vitro* we performed T cell proliferation assays to explore the immunomodulatory potential of iBreg after 7 days of MSC-B cell coculture. T cells isolated from same donor Tonsils were cocultured for 4 days in the presence of iBreg. iBreg significantly reduced polyclonal T cell proliferation in a dose dependent manner. This effect was clearly observed at 1:2 T cell to B cell ratios compared to activated B cells, but was not maintained at lower iBreg ratios (**Figures 2A, B**).

To investigate whether iBreg showed a stable phenotype, we explored the effect of different B cell stimulatory molecules added to MSC-B cell cocultures at day 7. To this end, iBreg were recovered and four different Breg polarizing stimuli were added to iBreg in the absence of MSC: 1) TCL stimuli (IL-2, CD40 agonist and anti-IgM), 2) CpG and 3) LPS (**Figure 2C**). While iBreg under unstimulated, LPS and CPG stimulated conditions, remained phenotypically stable, TCL stimulated iBreg showed a significant reduction on the percentage of transitional B cells.

### iBreg have high and stable IL-10^+^/TNFα^+^ cell ratios that allow the sorting of IL-10^+^ populations by flow cytometry for downstream analysis

To date, Breg studies define IL-10 as the most representative Breg cell marker and the hallmark of Breg functionality in humans. Thus, we aimed to characterize IL-10 expression on our iBregs produced cells.

**Figure 3:**
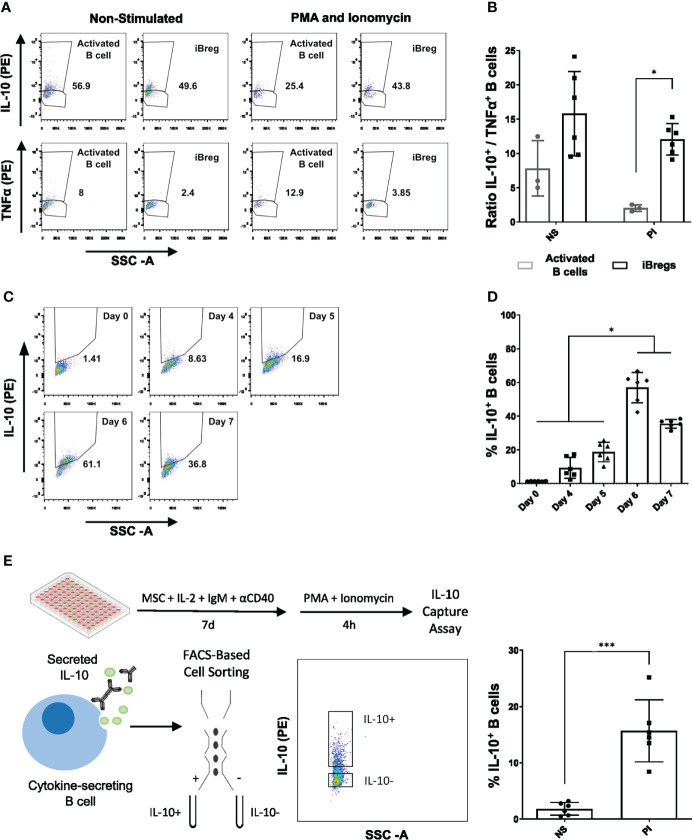
iBreg show high IL-10^+^/TNFα^+^ cell ratios after day 6 of culture, that remain constant after 4h PMA and Ionomycin stimuli. IL-10 capture assay was performed and used for downstream IL-10^+^ and IL-10^-^ flow cytometry associated cell sorting showing optimal results in PMA and Ionomycin samples. **(A)** Representative FACS plots showing the percentage of IL-10 and TNFα positive B cells in Activated B cell and iBreg conditions. **(B)** Ratio of IL-10^+^ and TNFα^+^ B cells after 7-day culture by intracellular staining after 4h stimulation with PMA and Ionomycin (PI) or without further stimulation (NS). In PI samples only iBreg retain high ratio IL-10^+^/TNFα^+^, showing higher phenotypical stability and regulatory cytokine profile, which was comparable to NS iBreg allowing for the use of PI stimuli for downstream applications. **(C)** Representative FACS plots showing the percentage of IL-10 positive iBreg at days 0, 4, 5, 7 of culture **(D)** Analysis of the percentage of IL-10^+^ B cells over 7-day culture performing 4h PMA and Ionomycin stimulation and intracellular staining. iBreg show a significant increase of IL-10^+^ B cells after day 6, suggesting optimal IL-10 expression at day 6 and 7 **(C)** Graphical protocol for IL-10 capture assay and the associated FACS-based cell sorting of IL-10^+^ and IL-10^-^ populations **(E)** Percentage of IL-10^+^ cells by flow cytometry in sorted samples. A total of 6 samples were sorted in each condition. PI samples showed high percentages of IL-10^+^ cells that allowed for transcriptomic analysis. For figures A and B Data shows at least three independent experiments in each group. Cells from 2 MSC donors and 3 Tonsil donors were used. Figure D represents per condition 6 sorted samples, performed in three batches of 2 samples. Error bars represent SD. Two-way ANOVA, One-way ANOVA or non-parametric T-test was performed to determine statistical significance. *p < 0.05, ***p < 0.001.

At day 7, intracellular staining revealed that 40% of iBreg expressed IL-10, while only 4% were positive for TNFα staining. Although slightly increased, IL-10^+^/TNFα^+^ ratio in iBregs did not significantly differ from activated B cells (**Figures 3A, B**). In contrast, when PMA and Ionomycin (PI) boost was added to cells before intracellular staining, a significant reduction of IL-10^+^/TNFα^+^ ratio was observed in activated B cells, associated to an increase of TNFα^+^ cells. In contrast, iBreg maintained a similar profile observed in non-PI iBreg conditions (**Figure 3B**). Of note, iBreg significantly increased IL-10^+^/TNFα^+^ ratio compared to PI-boosted activated B cells. Moreover, we analyzed intracellular IL-10 expression in MSC-B cell coculture over 7-day period performing 4h PI-boosting at each timepoint. In these conditions, IL-10 was significantly increased at day 6 and 7 compared to previous time-points (**Figures 3C, D**). These results indicate that a 4h PI stimulation did not significantly alter the cytokine profile of iBreg, while it promoted proinflammatory cytokines in activated B cells.

To better characterize iBreg populations obtained in our MSC-B cell coculture conditions at day 7, we sorted iBreg populations according to IL-10 expression (**Figure 3E**). A total of 6 iBreg samples from 3 different tonsils and 2 MSC donors were sorted into IL-10^+^ and IL-10^-^ fractions. Only iBreg PI-boosted samples allowed recovering enough number of IL10^+^ B cells for further analyses. RNA was extracted from sorted samples and analyzed, showing ideal yields and excellent integrity (**Supplementary Table 1**) for transcriptomic analysis.

### IL-10^+^ iBreg are not a distinct B cell functional subset, while IL-10^-^ iBreg are enriched in extracellular matrix and immune regulation genes

**Figure 4:**
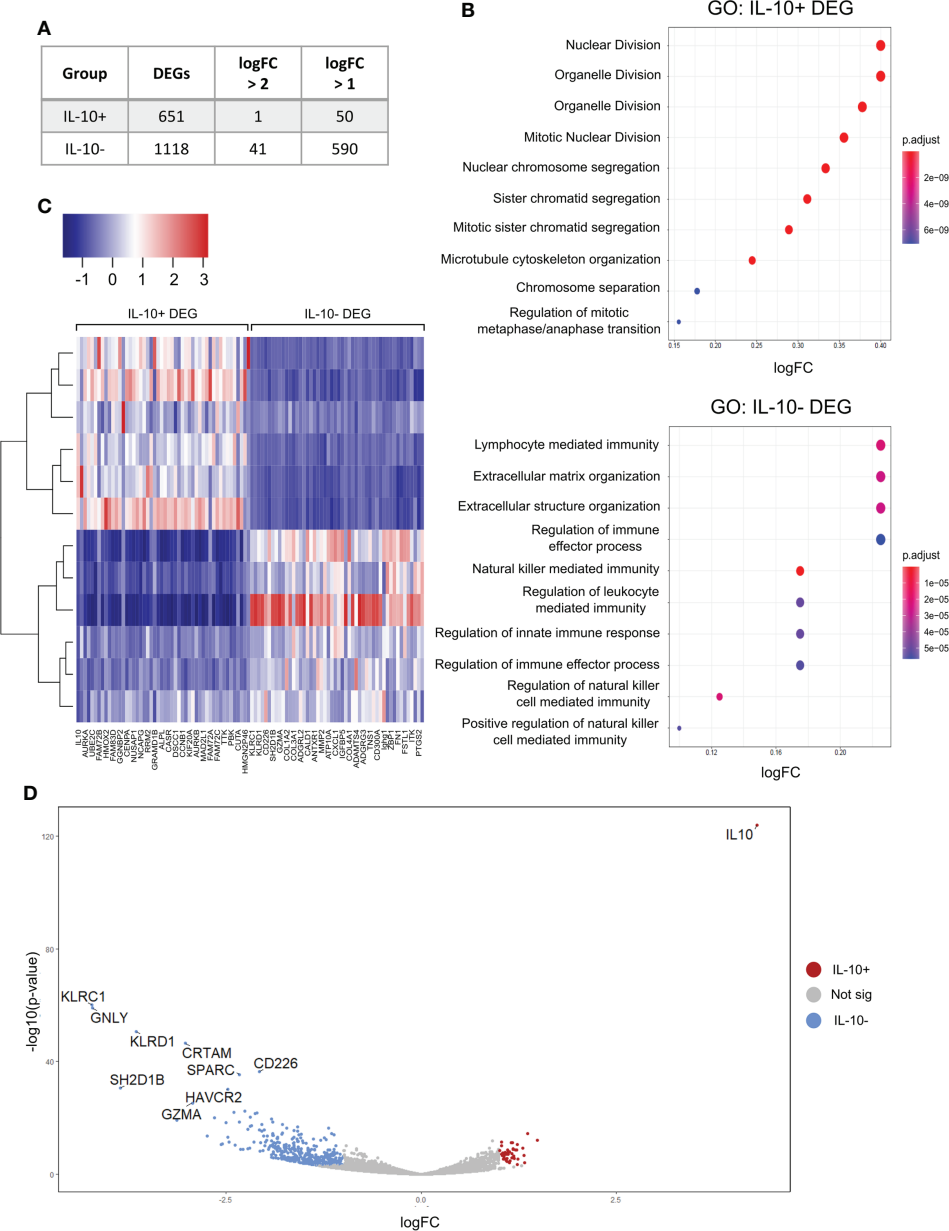
RNA-seq analysis of differentially expressed genes (DEG) shows an increased abundance of IL-10- DEG that are involved in extracellular matrix organization and immune regulation. IL-10+ are mainly involved in cellular division and activation. **(A)** Table shows number of DEG in both groups. We observed a higher relative abundance of IL-10^-^ DEG **(B)** Gene ontology analysis of biological processes in IL-10^+^ and IL-10^-^ DEG. IL-10^+^ DEG are enriched in cellular division processes suggesting that IL-10^+^ iBreg populations do not constitute a particular Breg population. IL-10^-^ DEG are associated to immune modulation and extracellular matrix organization, suggesting a potential association to Breg functionality and elucidating a possible link between extracellular matrixes and iBreg function. **(C)** Heatmap of top 20 DEG genes from both groups. **(D)** Volcano plot of genes identified by RNA-seq. Top 10 significant DEG are named on the plot. IL-10^-^ DEG show higher fold changes and p-values and constitute potential Breg biomarkers, while IL-10 is the only gene identified in IL-10^+^ populations with higher significance and fold change. Colored dots represent significant genes (p-value < 0.05) with a logFC value of ± 1.

RNA-seq analysis of sorted IL-10^+^ and IL-10^-^ iBreg successfully identified over 1700 genes were differentially expressed among both populations (**Figure 4A**), and showed distinct expression between groups (**Figure 4C**), that discriminated both populations.

First, we studied IL-10^+^ B cells differentially expressed genes (DEG). Gene ontology analysis of biological processes by gene ontology identified enrichment in processes only related to cellular division and activation (**Figure 4B**), and no genes related to immune regulation, nor related to Breg function, except for IL-10 expression.

Unexpectedly, we observed higher abundances and log2FC values of expressed genes in the IL-10^-^ DEG, when stablishing a cut-off value of ±1 log2FC. In addition, IL-10^-^ DEG were enriched in processes related to the generation and organization of the extracellular matrix (**Figure 4B**). Genes identified in previous works as Breg markers were differentially expressed in the IL-10^-^ iBreg DEG, such as GMZB, FASL, THSB1, IL1RN, CD1c, TIGIT or CD73 (**Figure 4D**, and **Supplementary Figure 4**). Other Breg biomarkers described in the literature were identified by RNA-seq, but their expression was stable in both groups (**Supplementary Figure 4**).

### MSC induce extracellular matrix genes that are upregulated in iBreg when compared to activated B cells

**Figure 5:**
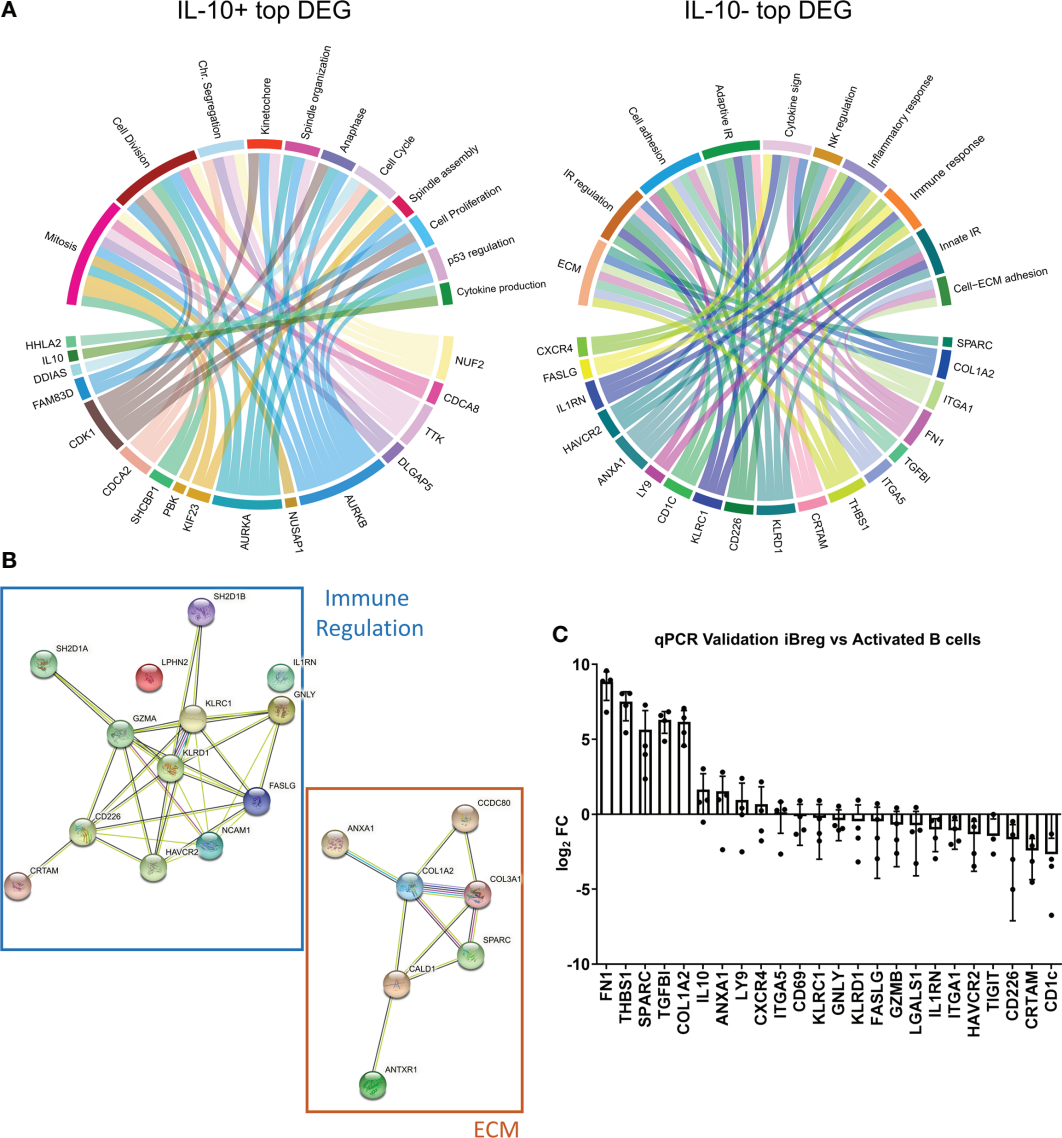
IL-10^-^ DEG involved in extracellular matrix organization are significantly upregulated in total iBreg when compared to activated B cells **(A)** Chord diagrams show biological processes of the top 20 DEG according to adjusted p-value, recapitulating similar biological processes to those identified in analysis involving all DEG. **(B)** Protein interactions of top DEG showing clear aggrupation in two functional networks of immune regulation and extracellular matrix genes. **(C)** qPCR validation of identified cell markers comparing expression between iBreg and activated B cells. Markers involved in extracellular matrix organization have clear distinct expressions on iBreg, while immune related markers have similar expressions or are downregulated in iBreg. Positive logFC values represent higher expression in iBreg compared to activated B cells. Cells from 2 MSC donors and 4 Tonsil donors were used for experiments.

To identify potential Breg biomarkers, we took a closer look at DEG with top statistical significances (**Supplementary Table 2**). Representation of the top 20 significant genes biological processes (**Figure 5A**) recapitulated similar overarching processes in both IL10^+^ and IL10^-^ groups compared to the whole list of DEG. IL-10^+^ genes were involved in cell processes such as cell activation and/or mitosis, with very few genes being involved in immune regulation. However, none of them were particularly interesting as candidates for downstream analysis due to heavily skewed expression among samples. In contrast, IL-10^-^ genes showed promising potential due to high and stable expressions across cell donors. Consistent with this data, IL-10^-^ DEG involved in extracellular matrix and immune regulation were associated to functional protein networks, as we could find several mediators of specific pathways simultaneously expressed, composing two clearly defined and independent functional networks (**Figure 5B**). These findings may suggest an enrichment in these particular pathways that may indicate an iBreg mechanism of action.

To validate our RNA-seq results, we further analysed gene expression by qPCR. First, we reproduced RNA-seq results comparing gene expression of 25 DEG genes in sorted IL-10^+^ and IL-10^-^ iBreg (data not shown). To further verify the biomarker potential of these genes, we compared gene expression between total non-sorted iBreg samples and *in vitro* activated B cells (**Figure 5C**). Nine out of the 24 genes tested had a differential expression in iBreg samples, while the others were overrepresented in activated B cells, or had similar expressions in both populations. Genes with a significant higher expression in iBreg compared to activated B cells included mostly those involved in the aforementioned extracellular matrix processes, *FN1, THSB1, SPARC, TGFBI, COL1A2, IL-10, ANXA1, LY9 and CXCR4*. Overall, our results suggest that MSC induce B cells to express genes involved in extracellular matrix organization, and to generate rich extracellular matrixes, as seen by the increase of fibronectin (*FN1*) and collagens (*COL1A2*) in addition to others not tested by qPCR. Furthermore, upregulated genes such as *TGFBI*, *THSB1* and the aforementioned extracellular matrix genes could be related to TGF-β mediated signalling.

### iBreg inhibition of T cell proliferation is independent of IL-10 direct effect

**Figure 6:**
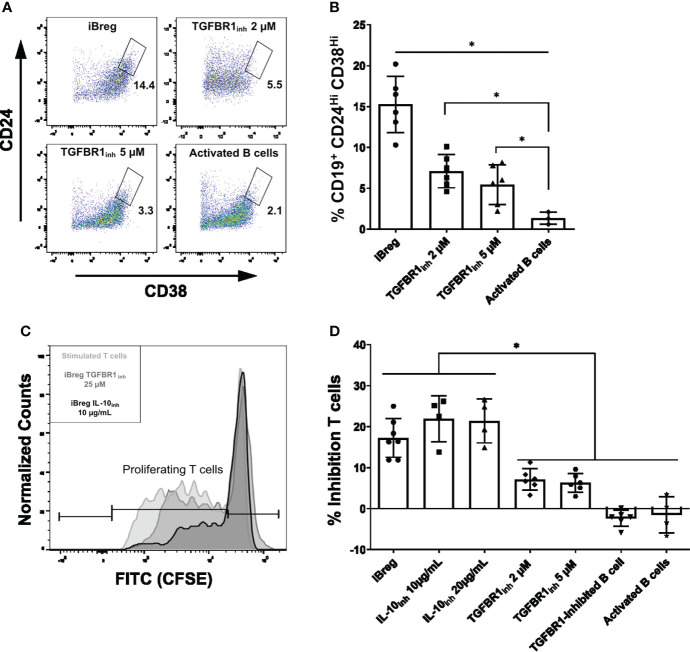
iBreg inhibition of T cell proliferation is independent of IL-10 effect on T cells. **(A)** Representative FACS plots showing the percentage of Transitional B (CD19^+^CD24^Hi^CD38^Hi^) cells from alive B cells (CD19^+^7AAD^-^). **(B)** Summarized data for the frequency of Transitional B cells in iBreg. TGFBR1 inhibitors significantly reduce transitional B cells induction by MSC, but are not comparable to activated B cells levels, highlighting a partial inhibition of MSC effect. **(C)** Histogram representing CFSE expression on T cells. Lighter grey represents stimulated T cell. Light grey and dark grey represent, stimulated T cells coculture with iBreg at 1:2 T to B cell ratios in the presence of an IL-10 neutralizing antibody or a TGFBR1 inhibitor, respectively. **(D)** Bar graph representing reduction of proliferating T cells for each condition compared to stimulated T cells controls. iBreg and iBreg samples with IL-10 neutralizing antibodies (IL-10_inh_ 10 and 20 µg/mL) show equal inhibition of T cell proliferation, which is significantly increased when compared to activated B cells. iBreg samples with TGFBR1 inhibitor (TGFBR1_inh_ 2 and 5 µM) show reduced suppression capabilities, while not being significantly different from activated B cells and B cells from MSC cocultures that were cocultured in the presence of inhibitor from day 0 (TGFBR1-inhibited B cells). Data shows at least three independent experiments in each group. Cells from 2 MSC donors and 3 Tonsil donors were used. Error bars represent SD. One-way ANOVA was performed to determine statistical significance. *p < 0.05.

Finally, we investigated whether IL-10 and TGF-β were direct mediators of iBreg regulatory capabilities and iBreg induction, as IL-10^+^ iBreg did not show any trait related to immune regulation when compared to IL-10^-^, and TGF-β related genes were upregulated in IL-10^-^ populations. First, we performed 7-day MSC-B cell coculture in the presence of LY-364947, a TGFBR1 inhibitor (**Figures 6A, B**). The percentage of Transitional B cells was significantly lower in the presence of the TGF-β inhibitor (15.2% vs 7.09% and 5.45% for iBreg, 2 µM and 5 µM, respectively), which could be attributed to TGF-β from MSC mediating transitional B cell induction. However, the percentage of transitional B cells in TGFBR1 inhibited B cells was significantly higher than in activated B cells, thus, TGF-β or TGFBR1 independent MSC mediators may also contribute to this effect. Second, we performed T cell proliferation assays to analyse iBreg inhibitory capacity in the presence of an IL-10 neutralizing antibody and the same TGFBR1 inhibitor. iBreg inhibited T cell proliferation even in the presence of two different concentrations of IL-10 neutralizing antibodies (**Figures 6C, D**). iBreg inhibition of T cell proliferation was statistically significant when compared to activated B cells either in the presence or absence of IL-10 blocking antibodies. However, TGFBR1 inhibition significantly reduced iBreg supression of T cell proliferation (17.28 vs 7.1 and 6.3% of inhibition for iBreg, 2 µM and 5 µM, respectively). Moreover, B cells cultured in the presence of 2 µM TGFBR1 inhibitor in MSC-B cell cocultures (TGFBR1_inh_ 2 µM B cell condition, **Figure 6B**) showed no suppression of T cell proliferation, and were not significantly different from activated B cells (TGFBR1-inhibited B cell condition, **Figure 6D**).

## Discussion

Breg have been extensively studied in the literature due to their association with tolerance in kidney transplanted patients (36, 37) and autoimmune diseases (9). However, there is a lack of unique defining human Breg markers that limits their clinical application. The canonical view on human Breg was originally focused on IL-10 as the gold standard to discern regulatory B cells, however, Breg research has diversified in recent years, transitioning from the study of Breg as a single subset, to the association of different markers to Breg across all B cell subsets (5–8).

In this work, we aimed to induce and characterize Breg by culturing B cells with mesenchymal stromal cells (MSC). MSC have been thoughtfully studied and used for their regulatory and regenerative capabilities. Nevertheless, MSC regulatory effect is not enough to induce Breg, as co-stimulation by T helper action and/or BCR, TLR and CD40 activation are necessary for *in vitro* studies. This need for cell activation to induce Breg is in line with the reported increase of the number of Bregs in response to inflammation (38). Furthermore, MSC regulatory effect is also boosted by inflammatory conditions (39). Thus, B cell-MSC interaction might constitute an *in vitro* model that better recapitulates Breg expansion in response to inflammatory conditions to promote tissue homeostasis, if the microenvironment possesses a correct balance of regulatory and proinflammatory components.

In this study, we started by culturing B cells with MSC, soluble IL-2, anti-IgM and CD40 agonist (T cell-like stimulation). We found MSC and T cell-like stimulating factors to allow for the expansion of moderate numbers of IL-10 producing iBreg, enriched in transitional B cell populations, with no associated TNFα induction. In agreement with previous descriptions of MSC effect on B cells, most B cells maintained a naïve B cell phenotype, and were able to suppress T cell proliferation.

Given the importance of IL-10^+^ Breg populations, we first determined their intracellular expression without further stimulation compared with a 4h PMA and Ionomycin (PI) boost. Overall, iBreg showed high IL-10^+^/TNFα^+^ B cell ratios, that were not altered by PI boost. We sorted IL-10^+^ and IL-10^-^ iBreg by performing a modified IL-10 capture assay with flow cytometry associated cell sorting to study differential gene expression by RNA-seq. Our major unexpected finding was that IL-10^+^ iBreg populations bear no enrichment in regulatory markers, while IL-10^-^ iBreg populations were enriched in regulatory and extracellular matrix genes. As a proof of concept, we performed T cell suppression assays and found that IL-10 neutralization had no effect over iBreg immunomodulatory capabilities.

So far, IL-10 was considered crucial for MSC-induced regulatory B cell populations, however, IL-10 has been proven a double edge sword in human Breg *in vitro* studies (40). IL-10 is a regulatory cytokine highly expressed *in vivo* by regulatory subsets such as Treg, but it is also expressed in B cells in response to cellular activation, which might be artefactually overused to allow *in vitro* culture, compared to the degree of stimulation present *in vivo.* Moreover, IL-10 production *in vitro* is associated to TRL ligation (41) which could explain the absence of *bona fide* IL-10^+^ Breg cells in our culture setting, as we did not use TLR ligands for B cell activation. Therefore, we cannot conclude that IL-10 is associated to Breg in our experiment settings, thus, MSC-B cell coculture may be an interesting platform for the study of IL-10 independent Breg mechanisms. These findings are in line with other Breg *in vitro* culture systems describing secreted factors such as GZMB as the mechanism of action of Breg, independently of IL-10 (42). In fact, we have studied the expression of GZMB in the RNAseq analysis and found it differentially expressed in IL10- B cells, although it was no overexpressed in iBregs compared to activated B cells (**Figure 5D**). In this line, markers and potential key mediators for Breg identified in the literature [reviewed in (43)] such as PD-L1, IL-35, IDO among others have been interrogated in the RNAseq data. The expression of these genes has not been found differentially expressed between the two groups of analysis.

In this study, we identified immune related genes, in addition to extracellular matrix (ECM) organization markers, as being over-represented in sorted IL-10^-^ iBreg. We compared expression of this markers by qPCR between iBreg and *in vitro* activated B cells. We found ECM genes to be upregulated in iBreg, with substantial overexpression of Fibronectin, Collagen and Thrombospondin-1. In addition, upregulation of extracellular matrix and markers such as Thrombospondin-1 and TGFBI point at TGF-β, which is highly produced by MSC, as a crucial mediator of iBreg induction. In fact, inhibition of TGFBR1 in MSC-B cell cocultures partially abrogated transitional B cell induction. This effect was correlated to their T cell modulation capabilities, as inhibition of TGFBR1 in the MSC-B cell coculture fully inhibited B cell-mediated suppression of T cell proliferation (**Figure 6D**) rendering TGFBR1-inhibited iBregs comparable to activated B cells. MSC secretome is a major contributor to MSC therapeutic effect, and according to our *in vitro* results, TGF-β could be a key component for Breg induction. Moreover, inhibition of TGFBR1 was also directly involved in iBreg immunomodulatory action. Functional iBreg displayed a reduced suppression of T cell proliferation in the presence of TGFBR1 inhibitor (**Figure 6D**) showing that TGF-β is partially involved in iBreg mediated suppresion.

Classically, B cells studies have focused on antibody production, cellular costimulation, antigen presentation and cytokine production. However, MSC-B cell coculture highlights the importance of ECM regulation, which may be crucial for immune modulation in tissues and is omitted in most *in vitro* settings. ECM components orchestrate cell survival, activation, differentiation and proliferation, playing an important role in the modulation of tissue homeostasis and cellular crosstalk. ECM proteins found in IL-10^-^ iBreg, such as Fibronectin1, Thrombospondin1 and Laminin 4 (**Supplementary Figure 4**) have been described to modulate T cell activation and differentiation (44–48), and may be a major mediator of Breg immunomodulation.

B cells interact in lymph nodes with follicular T helper cell, but also stromal cells such as follicular reticular cells, that will stablish the ECM. Upon circulation and migration to inflammatory environments, B cells will be challenged by a particular ECM composition that will influence immune responses. Whether Breg function is dictated by ECM composition or Breg have the capability to influence “foreign” ECM environments remains to be answered in future studies. In addition, a better understanding of such markers would allow for the better monitoring of inflammatory responses in MSC cell therapies.

MSC-Breg induction elucidates B cell populations with characteristic features that open up the study of novel Breg subsets characterized by the generation of extracellular matrixes and immune modulation independently of IL-10 direct action. In addition, TGF-β mediates MSC-induced Breg generation and immunomodulatory capabilities. These findings build the basis for further mechanistic studies, required to better understand the biology and function of Breg.

## Data availability statement

The datasets presented in this study can be found in online repositories. The names of the repository/repositories and accession number(s) can be found below:

NCBI under accession ID: PRJNA858585

## Ethics statement

All participants and/or their legal tutor gave written informed consent for participation. The study protocol followed the principles of the Declaration of Helsinki and was approved by the Clinical Research Ethics Committee of our institution (Comitè Ètic d’Investigació Clínica, Refs CEIC: PI-16-056).

## Author Contributions

SG: Designed the study, performed experiments, formal analysis, figure creation, data curation, and writing original draft. NS-H: Performed experiments, formal analysis and data curation. MC-S: Performed experiments and formal analysis. LC-P: Performed experiments and formal analysis. MM-F: Performed experiments. DG: Human sample and data curation. FB: Study design and formal analysis. MF: Designed the study, performed experiments, formal analysis, data curation. All authors interpreted, discussed the data and participated in reviewing and editing the draft. All authors contributed to the article and approved the submitted version.

## Funding

This work was supported in part by SGR program of Generalitat de Catalunya (2017-SGR-301 REMAR Group) and Instituto Carlos III project PI17/00335, integrated in the National R + D + I and funded by the ISCIII and the European Regional Development Fund. SG is supported by the Catalan Health department (“Departament de Salut”) in receipt of a grant from PERIS-PIF-Salut (SLT017/20/000158), MC-S is supported by a grant from ISCIII (FI20/00021), FB is a senior researcher from Germans Trias i Pujol Health Science Research Institute, supported by the Health Department of the Catalan Government (Generalitat de Catalunya) and MF is supported by ISCIII (MS19/00018), co-funded by ERDF/ESF, “Investing in your future”.

## Acknowledgments

We thank the Flow cytometry and the genomic departments of the IGTP for their valuable technical help and their contribution to the experiments.

## Conflict of Interest

The authors declare that the research was conducted in the absence of any commercial or financial relationships that could be construed as a potential conflict of interest.

## Publisher’s note

All claims expressed in this article are solely those of the authors and do not necessarily represent those of their affiliated organizations, or those of the publisher, the editors and the reviewers. Any product that may be evaluated in this article, or claim that may be made by its manufacturer, is not guaranteed or endorsed by the publisher.

## Supplementary material

Supplementary Figure 1 MSC differentiate to adipocyte and osteocyte cell types and express MSC surface markers. **(A)** Pictures from MSC multilineage differentiation assays. Left column shows adipocyte differentiation from donors 1 and 2. Right column shows osteocyte differentiation from donors 1 and 2. **(B)** Flow cytometry analysis of the percentage of positive cells for each MSC marker. Cells are positive for CD90, CD105 and CD73, and negative for CD45 and HLA-DR. Histograms show isotype control in red, and 4 MSC donors in different colors. Bottom right bar graph represents mean percentage of positive cells for each marker.

Supplementary Figure 2 Anti-IL-10 antibody neutralized recombinant IL-10 detection by ELISA. 100 and 50 pg/mL of recombinant IL-10 was detected by ELISA without anti-IL-10 antibody (-) or in the presence of 10 or 20 µg/mL of antibody. Two-way ANOVA was performed to determine statistical significance. ns (non-significant) p > 0.05, * p < 0.05, ** p < 0.01, *** p < 0.001, **** p < 0.0001.

Supplementary Figure 3 MSC induce regulatory B cells that are primarily enriched in transitional naïve B cells, with excellent cell viability and maintain low memory cell. **(A)** Flow cytometry analysis of the percentage of alive B cells, gated as cells CD19+ and 7AAD-. **(B)** Flow cytometry analysis of the percentage of naïve B cells, gated as cells CD27- and IgD+, in transitional B cell populations, gated as CD19+CD24HiCD38Hi. **(C)** Flow cytometry analysis of the percentage of memory switched B cells from total B cells, gated as cells CD19+CD27+IgD-. Error bars represent SD. Two-way ANOVA was performed to determine statistical significance. ns (non-significant) p > 0.05, * p < 0.05, ** p < 0.01, *** p < 0.001, **** p < 0.0001.

Supplementary Figure 4 Analysis of published Breg markers distribution between IL-10+ and IL-10- groups comparing normalize gene expression. Graphs include gene Name and p-value from the statistical analysis. Error bars represent SD. Non-parametric T-test was performed to determine statistical significance.
