# Supplementary figures and images for: Mesenchymal stromal cells induced regulatory B cells are enriched in extracellular matrix genes and IL-10 independent modulators
Source: Front Immunol. 2022 Sep 14;13:957797. doi: 10.3389/fimmu.2022.957797 (PMC9515545; doi:10.3389/fimmu.2022.957797)

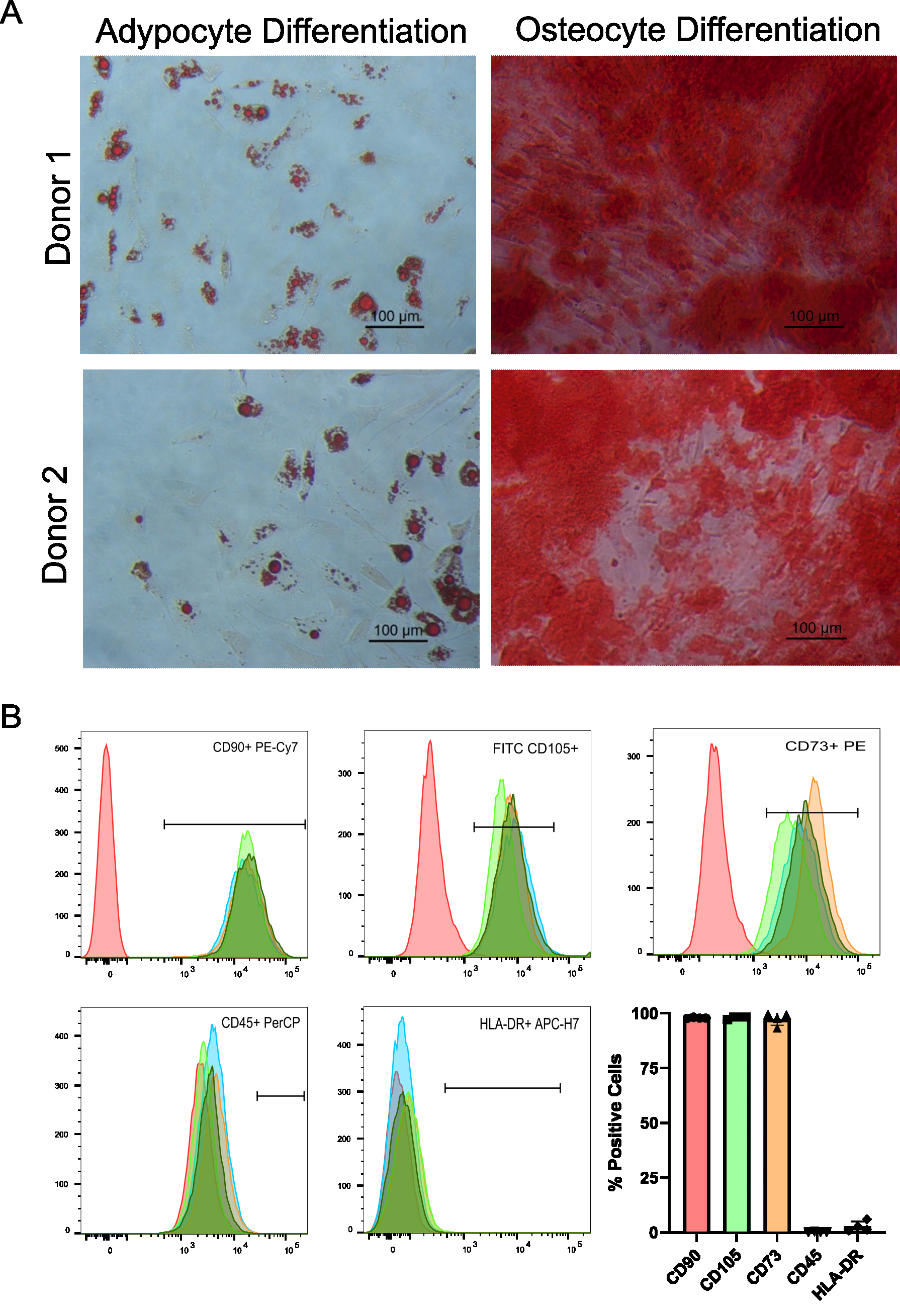

Supplement: Supplementary Figure 1 — MSC differentiate to adipocyte and osteocyte cell types and express MSC surface markers. (A) Pictures from MSC multilineage differentiation assays. Left column shows adipocyte differentiation from donors 1 and 2. Right column shows osteocyte differentiation from donors 1 and 2. (B) Flow cytometry analysis of the percentage of positive cells for each MSC marker. Cells are positive for CD90, CD105 and CD73, and negative for CD45 and HLA-DR. Histograms show isotype control in red, and 4 MSC donors in different colors. Bottom right bar graph represents mean percentage of positive cells for each marker. [file Image_1.tiff]

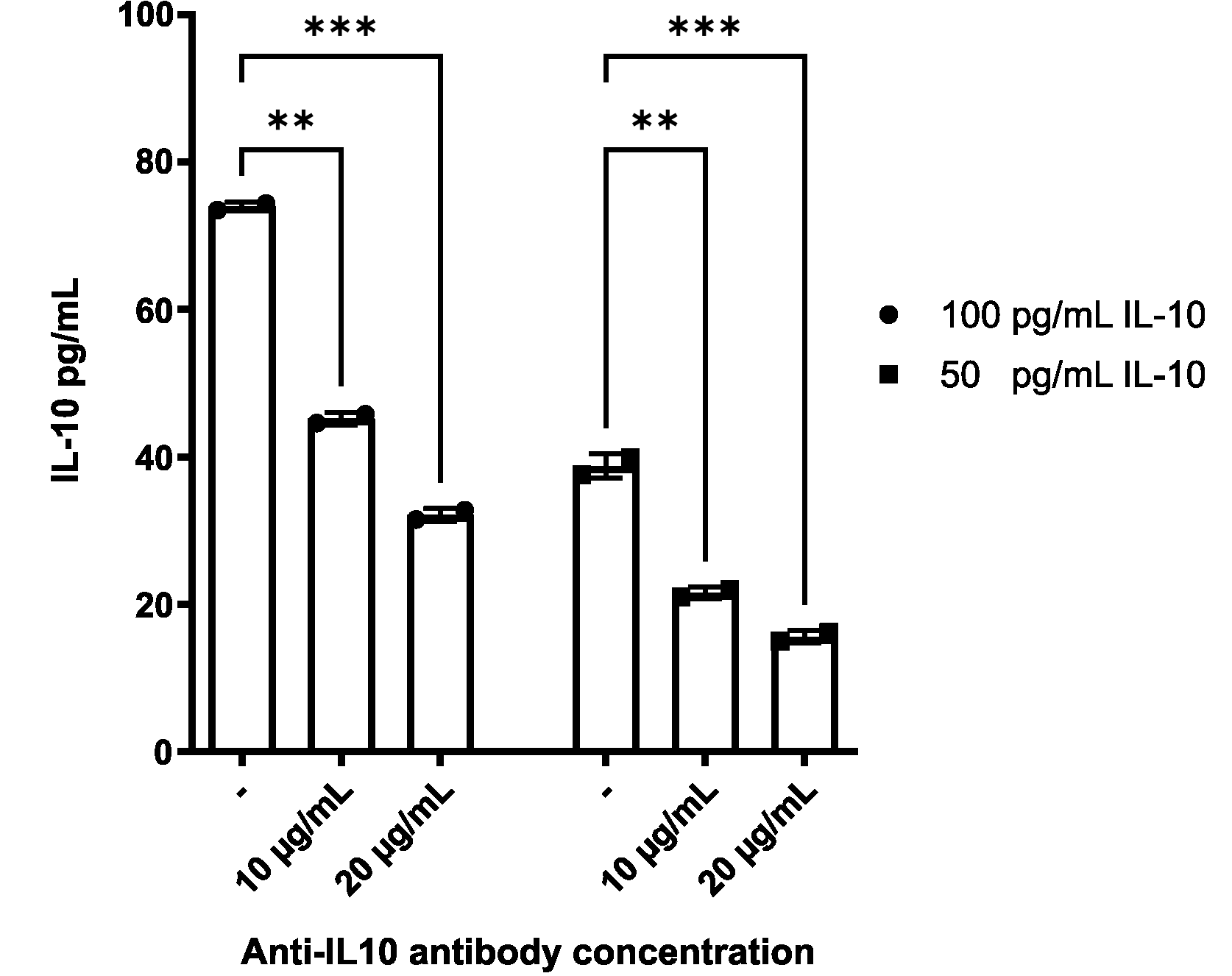

Supplement: Supplementary Figure 2 — Anti-IL-10 antibody neutralized recombinant IL-10 detection by ELISA. 100 and 50 pg/mL of recombinant IL-10 was detected by ELISA without anti-IL-10 antibody (-) or in the presence of 10 or 20 µg/mL of antibody. Two-way ANOVA was performed to determine statistical significance. ns (non-significant) p > 0.05, * p < 0.05, ** p < 0.01, *** p < 0.001, **** p < 0.0001. [file Image_2.tiff]

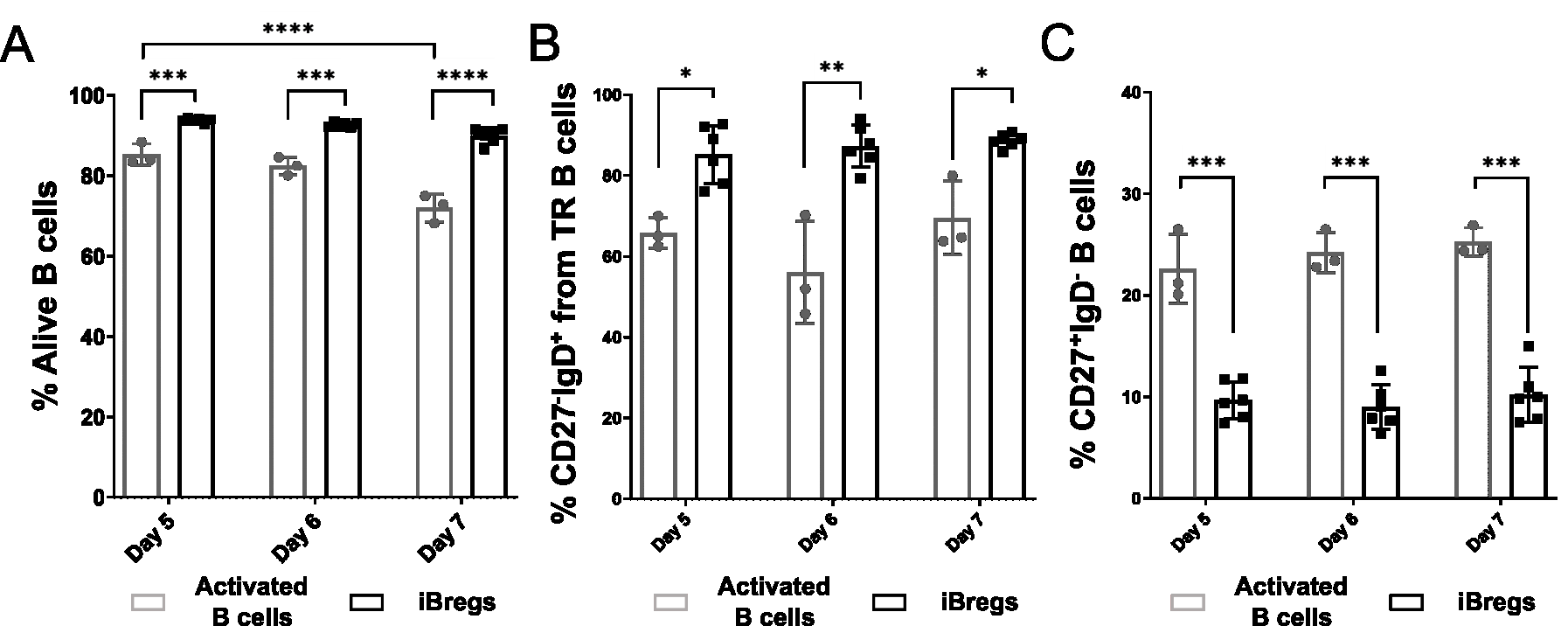

Supplement: Supplementary Figure 3 — MSC induce regulatory B cells that are primarily enriched in transitional naïve B cells, with excellent cell viability and maintain low memory cell. (A) Flow cytometry analysis of the percentage of alive B cells, gated as cells CD19+ and 7AAD-. (B) Flow cytometry analysis of the percentage of naïve B cells, gated as cells CD27- and IgD+, in transitional B cell populations, gated as CD19+CD24HiCD38Hi. (C) Flow cytometry analysis of the percentage of memory switched B cells from total B cells, gated as cells CD19+CD27+IgD-. Error bars represent SD. Two-way ANOVA was performed to determine statistical significance. ns (non-significant) p > 0.05, * p < 0.05, ** p < 0.01, *** p < 0.001, **** p < 0.0001. [file Image_3.tiff]

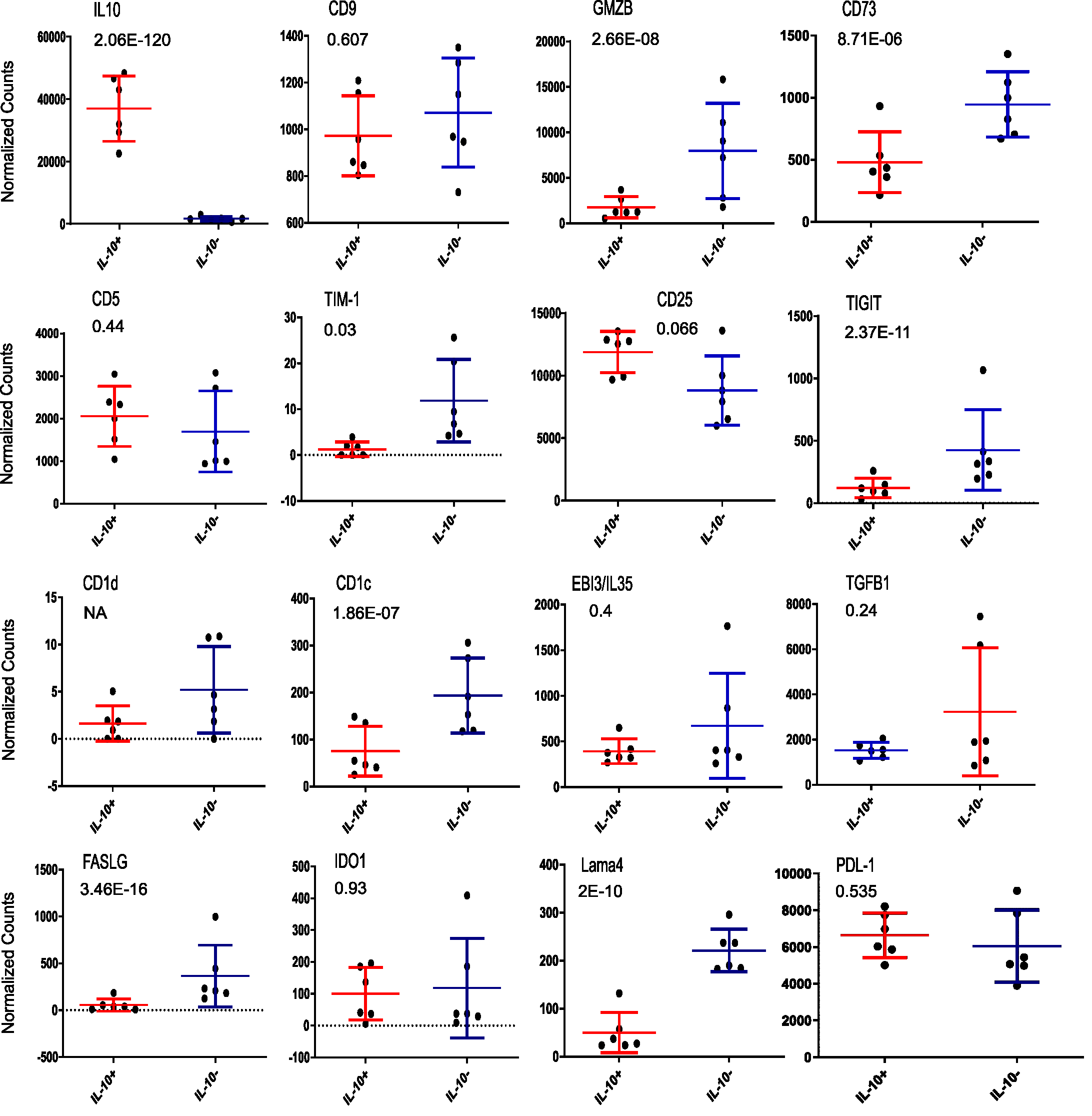

Supplement: Supplementary Figure 4 — Analysis of published Breg markers distribution between IL-10+ and IL-10- groups comparing normalize gene expression. Graphs include gene Name and p-value from the statistical analysis. Error bars represent SD. Non-parametric T-test was performed to determine statistical significance. [file Image_4.tiff]
