# Supplementary material for: Mesenchymal stromal cells induced regulatory B cells are enriched in extracellular matrix genes and IL-10 independent modulators
Source: Front Immunol. 2022 Sep 14;13:957797. doi: 10.3389/fimmu.2022.957797 (PMC9515545; doi:10.3389/fimmu.2022.957797)
Supplement: Supplementary file 5 [file Table_1.docx]

Supplementary Table 1. RNA samples obtained from Sorted iBreg IL-10^+/-^ samples

| B cell Donor | MSC donor | Sorted cells | | Total RNA (ng) | | RIN^e^ | |
| --- | --- | --- | --- | --- | --- | --- | --- |
| - | | IL-10+ | IL-10- | IL-10+ | IL-10- | IL-10+ | IL-10- |
| 1 | A | 112700 | 100000 | 436.8 | 286.68 | 9.2 | 9.5 |
| 2 | A | 99600 | 100000 | 969.9 | 452.4 | 9.2 | 9.4 |
| 3 | A | 90800 | 100000 | 624 | 384.8 | 9.5 | 9.5 |
| 1 | B | 100000 | 100000 | 780 | 237.9 | 9 | 9.4 |
| 2 | B | 100000 | 100000 | 240 | 193.18 | 9.2 | 9.5 |
| 3 | B | 84500 | 100000 | 332.8 | 296.4 | 9.1 | 9.3 |
