# Supplementary material for: Mesenchymal stromal cells induced regulatory B cells are enriched in extracellular matrix genes and IL-10 independent modulators
Source: Front Immunol. 2022 Sep 14;13:957797. doi: 10.3389/fimmu.2022.957797 (PMC9515545; doi:10.3389/fimmu.2022.957797)
Supplement: Supplementary file 6 [file Table_2.docx]

Supplementary Table 2. DEG analysis data from RNA-seq top 20 DEG from IL-10^+/-^ samples

| **IL-10^+^ top 20 DEG** | | | | **IL-10^-^ top 20 DEG** | | | |
| --- | --- | --- | --- | --- | --- | --- | --- |
| **Entrez Gene ID** | **Gene Symbol** | **Adjusted p Value** | **Log2(FC)** | **Entrez Gene ID** | **Gene Symbol** | **Adjusted p Value** | **Log2(FC)** |
| 3586 | IL10 | 2.06E120 | 4.304 | 3821 | KLRC1 | 6.89E57 | 4.223 |
| 11065 | AURKA | 1.36E12 | 1.362 | 10578 | GNLY | 5.34E56 | 4.214 |
| 1001 | CDCA2 | 1.83E10 | 1.487 | 3824 | KLRD1 | 9.19E48 | 3.655 |
| 6241 | CDK1 | 7.90E10 | 1.031 | 56253 | CRTAM | 1.02E43 | 3.025 |
| 891 | HHLA2 | 1.18E09 | 1.171 | 10666 | CD226 | 1.14E33 | 2.078 |
| 9077 | RRM2 | 1.50E09 | 1.152 | 6678 | SPARC | 9.17E33 | 2.330 |
| 81610 | FAM72D | 4.11E09 | 1.232 | 117157 | SH2D1B | 5.26E28 | 3.860 |
| 993 | PBK | 1.37E08 | 1.033 | 84868 | HAVCR2 | 1.06E27 | 2.476 |
| 57476 | DLGAP5 | 7.28E08 | 1.303 | 3001 | GZMA | 7.83E23 | 2.933 |
| 55355 | DDIAS | 1.08E07 | 1.181 | 4068 | SH2D1A | 7.15E20 | 2.260 |
| 890 | NUSAP1 | 1.39E07 | 1.178 | 1278 | COL1A2 | 1.12E19 | 2.405 |
| 983 | SPIC | 2.32E07 | 1.209 | 301 | ANXA1 | 1.88E19 | 2.107 |
| 7153 | FAM83D | 2.43E07 | 1.227 | 1281 | COL3A1 | 2.90E19 | 2.125 |
| 55143 | NUF2 | 2.78E07 | 1.107 | 151887 | CCDC80 | 4.88E18 | 2.190 |
| 79801 | ZNF492 | 4.86E07 | 1.005 | 23266 | ADGRL2 | 7.99E18 | 2.650 |
| 157313 | SHCBP1 | 8.32E07 | 1.130 | 4684 | NCAM1 | 6.30E17 | 3.134 |
| 83540 | AURKB | 1.23E06 | 1.079 | 800 | CALD1 | 2.25E16 | 2.331 |
| 9212 | KIF23 | 2.06E06 | 1.043 | 356 | FASLG | 3.46E16 | 2.499 |
| 55872 | CDCA8 | 2.09E06 | 1.097 | 84168 | ANTXR1 | 1.61E15 | 2.042 |
| 9493 | TTK | 2.48E06 | 1.042 | 3557 | IL1RN | 4.53E15 | 2.177 |
