# Supplementary material for: Mesenchymal stromal cells induced regulatory B cells are enriched in extracellular matrix genes and IL-10 independent modulators
Source: Front Immunol. 2022 Sep 14;13:957797. doi: 10.3389/fimmu.2022.957797 (PMC9515545; doi:10.3389/fimmu.2022.957797)
Supplement: Supplementary file 7 [file Table_3.docx]

**Supplementary Table 3. qPCR Primer Design**

| Gene | Sequence (5’-3’) | Template Strand | Primer Length | Tm (ºC) | % GC | Amplicon size (bp) |
| --- | --- | --- | --- | --- | --- | --- |
| FN1 | AACCTTGCTCCTGACAGCTC | Forward | 20 | 59.96 | 55.00 | 148 |
|  | TTGGTGGGCTGACATTCTCC | Reverse | 20 | 59.96 | 55.00 |  |
| THBS1 | GGACTCTGACGGCGATGGTC | Forward | 20 | 62.62 | 65 | 122 |
|  | ATCGGCGGAAATCGGTCTC | Reverse | 19 | 59.93 | 57.89 |  |
| SPARC | TGGCGAGTTTGAGAAGGTGT | Forward | 20 | 59.53 | 50 | 132 |
|  | CAAGGCCCGATGTAGTCCAG | Reverse | 20 | 60.18 | 60 |  |
| TGFBI | TGCTCCCACAAATGAAGCCT | Forward | 20 | 59.89 | 50 | 127 |
|  | GCCTCCGCTAACCAGGATTT | Reverse | 20 | 60.11 | 55 |  |
| COL1A2 | CTGGTCTCGGTGGGAACTTT | Forward | 20 | 59.6 | 55 | 75 |
|  | TTAAGCCCATTGGTCCAGGG | Reverse | 20 | 59.67 | 55 |  |
| IL10 | CGAGATGCCTTCAGCAGAGT | Forward | 20 | 59.82 | 55 | 189 |
|  | CGCCTTGATGTCTGGGTCTT | Reverse | 20 | 60.04 | 55 |  |
| ANXA1 | GTGAAGTGCGCCACAAGCAA | Forward | 20 | 62.34 | 55 | 76 |
|  | GGCGAGTTCCAACACCTTTCA | Reverse | 21 | 61.08 | 52.38 |  |
| LY9 | CTTCCTGCTCATGGGACTAAGA | Forward | 22 | 59.23 | 50 | 140 |
|  | TGGGACCAATCCAGATGACG | Reverse | 20 | 59.46 | 55 |  |
| CXCR4 | GAAACCCTCAGCGTCTCAGT | Forward | 20 | 59.68 | 55 | 154 |
|  | AGTAGTGGGCTAAGGGCACA | Reverse | 20 | 60.55 | 55 |  |
| ITGA5 | GGCTTCAACTTAGACGCGGAG | Forward | 21 | 61.33 | 57.14 | 140 |
|  | TGGCTGGTATTAGCCTTGGGT | Reverse | 21 | 61.19 | 52.38 |  |
| CD69 | CTCATTGCCTTATCAGTGGGC | Forward | 21 | 59.05 | 52.38 | 104 |
|  | TAGCCAACCCAGTCCTCAGA | Reverse | 20 | 59.88 | 55 |  |
| KLRC1 | AGACTAACCTGGCCTCTCCA | Forward | 20 | 59.59 | 55 | 71 |
|  | TGATGTCAGGGACTGTACTCTTC | Reverse | 23 | 59.24 | 47.83 |  |
| GNLY | CAACCCAGGTCTGGTCTTCT | Forward | 20 | 58.94 | 55.00 | 128 |
|  | TGGTCAACAGGTCACCCTGG | Reverse | 20 | 61.71 | 60.00 |  |
| KLRD1 | TCTCCAGCTCAGCTTCAACAA | Forward | 21 | **59.58** | **47.62** | 115 |
|  | CAGAGTGGTCTTAAACACTGCC | Reverse | 22 | 59.19 | 50.00 |  |
| FASLG | ACAGAAGGAGCTGGCAGAAC | Forward | 20 | 59.96 | 55.00 | 102 |
|  | CCTTGAGTTGGACTTGCCTGT | Reverse | 21 | 60.48 | 52.38 |  |
| GZMB | GATCATCGGGGGACATGAGG | Forward | 20 | 59.68 | 60.00 | 158 |
|  | TGACATTTATGGAGCTTCCCCA | Reverse | 22 | 59.42 | 45.45 |  |
| LGALS1 | CGCTAAGAGCTTCGTGCTGAAC | Forward | 22 | 62.1 | 54.55 | 186 |
|  | CACACCTCTGCAACACTTCCAG | Reverse | 22 | 61.64 | 54.55 |  |
| IL1RN | TCCGCAGTCACCTAATCACTC | Forward | 21 | 59.52 | 52.38 | 110 |
|  | AACATCCCAGATTCTGAAGGC | Reverse | 21 | 57.99 | 47.62 |  |
| ITGA1 | CCGAAGAGGTACTTGTTGCAGC | Forward | 22 | 61.75 | 54.55 | 107 |
|  | GGCTTCCGTGAATGCCTCCTTT | Reverse | 22 | 63.42 | 54.55 |  |
| HAVCR2 | TGTGCCTAACAGAGGTGTCC | Forward | 20 | 59.31 | 55.00 | 118 |
|  | TCCACTTCTGAGGACCTTGT | Reverse | 20 | 57.90 | 50.00 |  |
| TIGIT | GGTCCTAGAAAGCTCAGTGGC | Forward | 21 | 60.41 | 57.14 | 122 |
|  | TTCTAGTCAACGCGACCACC | Reverse | 20 | 60.04 | 55 |  |
| CD226 | GCCCTATGCTGAGAGGGTTT | Forward | 20 | 59.45 | 55.00 | 180 |
|  | GCTGCCTCAAAACTATCTGACTG | Reverse | 23 | 59.63 | 47.83 |  |
| CRTAM | GCTTCTTCATCACTCGGCCA | Forward | 20 | 60.39 | 55.00 | 145 |
|  | CTTGAAAGGAGTTGCCAGCAC | Reverse | 21 | 60.00 | 52.38 |  |
| CD1C | GGGTGACATGGATGCGGAAT | Forward | 20 | 60.47 | 55.00 | 197 |
|  | GGAAAAGTGGTGTCCCCAGTAG | Reverse | 22 | 60.55 | 54.55 |  |
